# A new Cretaceous Metatherian mammal from Henan, China

**DOI:** 10.7717/peerj.896

**Published:** 2015-04-14

**Authors:** Shundong Bi, Xingsheng Jin, Shuo Li, Tianming Du

**Affiliations:** 1 Key Laboratory of Vertebrate Evolution and Human Origins of Chinese Academy of Sciences, Institute of Vertebrate Paleontology and Paleoanthropology, Chinese Academy of Sciences, Beijing, China; 2 Department of Biology, Indiana University of Pennsylvania, Indiana, PA, United States of America; 3 Zhejiang Museum of Natural History, Hangzhou, Zhejiang Province, China

**Keywords:** Mammal, Henan, Tooth formula and replacement pattern, Upper Cretaceous, Basal metatherian

## Abstract

We report a new deltatheroidan mammal from the Upper Cretaceous of Henna, China. The new taxon, *Lotheridium mengi*, is based on a nearly complete skull and associated lower jaws with full adult dentition. Deltatheroidans are known mostly from fragmentary specimens from Asia and North America. Previous views on deltatheroidan relationships were diverse, but recent studies favored their metatherian affinity. The new specimen represents the most complete skull known for deltatheroidans and provides additional evidence that deltatheroidans already had the distinctive metatherian dental formula and replacement pattern and several other derived metatherian features, supporting the metatherian status for this clade. The new species also indicates that deltatheroidan mammals were more diverse and had broader geographical distributions than previously thought.

## Introduction

Deltatheroida is a clade of small basal metatherian mammals known from the Cretaceous of Asia and North America that have significant implications on the origin of therians and the timing of the eutherian-metatherian diversification. Deltatheroidans have previously been referred as eutherians (Gregory & Simpson, 1926; McKenna, Mellett & Szalay, 1971; Simpson, 1928; Van Valen, 1966), or a stem group of boreosphenidans outside of Eutheria and Metatheria (Butler & Kielan-Jaworowska, 1973; Cifelli, 1993a; Cifelli, 1993b; Fox, 1974; Fox, 1975; Fox & Naylor, 2006; Kielan-Jaworowska, 1975; Kielan-Jaworowska, Eaton & Bown, 1979). Although most recent studies favored the Deltatheroida as a basal clade within Metatheria (Averianov, Archibald & Ekdale, 2010; Davis, Cifelli & Kielan-Jaworowska, 2008; Kielan-Jaworowska & Nessov, 1990; Luo et al., 2003; Marshall & Kielan-Jaworowska, 1992; Muizon & Lange-Badré, 1997; Rougier, Wible & Novacek, 1998; Rougier, Wible & Novacek, 2004; Rougier, Davis & Novacek, 2015; Sánchez-Villagra et al., 2007; Szalay, 1994; Williamson, Brusatte & Wilson, 2014), its phylogenetic position remains controversial mainly owing to the fragmentary nature of the fossil record (Fox & Naylor, 2006; Kielan-Jaworowska, Cifelli & Luo, 2004). Until recently, deltatheroidans were known primarily from isolated teeth. Only *Deltatheridium* and *Deltatheroides* are known from partial skulls (Gregory & Simpson, 1926; Rougier, Wible & Novacek, 1998). Here we report a nearly complete skull with associated mandibles from the Upper Cretaceous of Henan, representing the most complete skull known for deltatheroidans. The fossil provides additional evidence on tooth formula and replacement pattern and craniodental morphologies of deltatheroidan mammals, lending support to their affinity with marsupials.

## Systematic Palaeontology

**Table:** 

Mammalia Linnaeus, 1758
Metatheria Huxley, 1880
Deltatheroida Kielan-Jaworowska, 1981
Deltatheridiidae (Gregory & Simpson, 1926)
*Lotheridium* gen. nov.

**Type species** *Lotheridium mengi* gen.et sp. nov.

**Diagnosis** As for the type species

**Etymology** Lo-, in reference to the prefecture of Luoyang, where the fossil was recovered, and -theridium, from the Greek theridion, meaning small beast.

*Lotheridium mengi* gen.et sp. nov.

**Figure 1:**
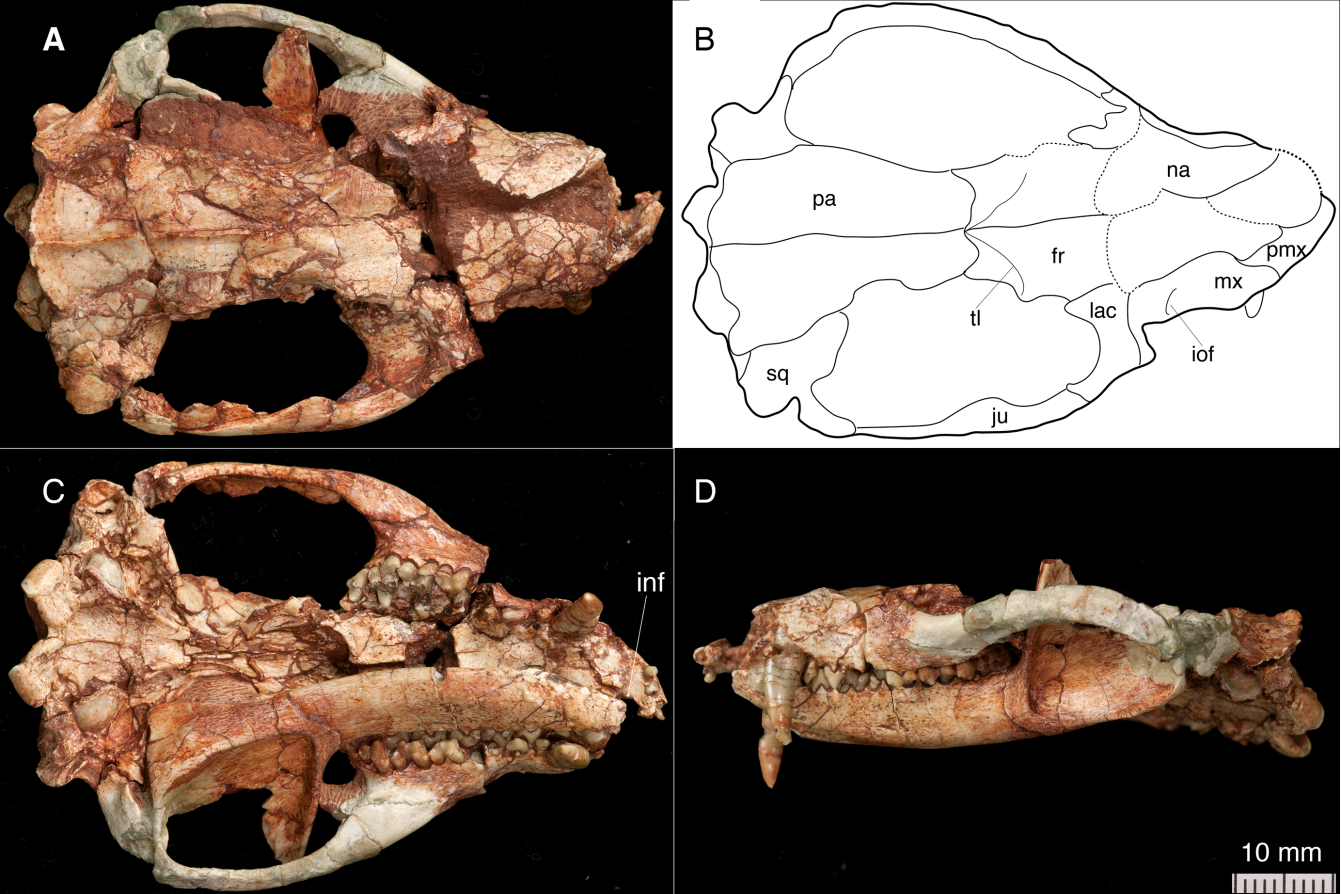
The skull and left mandible of *Lotheridium mengi* (ZMNH M9032). (A) The skull in dorsal view. (B) Line drawing of the skull in dorsal view. (C) The skull and left mandible in ventral view. (D) The skull and left mandible in lateral view. fr, frontal; inf, incisive foramen; iof, infraorbital foramen; ju, jugal; lac, lacrimal; mx, maxillae; na, nasal; pa, parietal; pmx, premaxilla; sq, squamosal; tl, temporal line.

**Figure 2:**
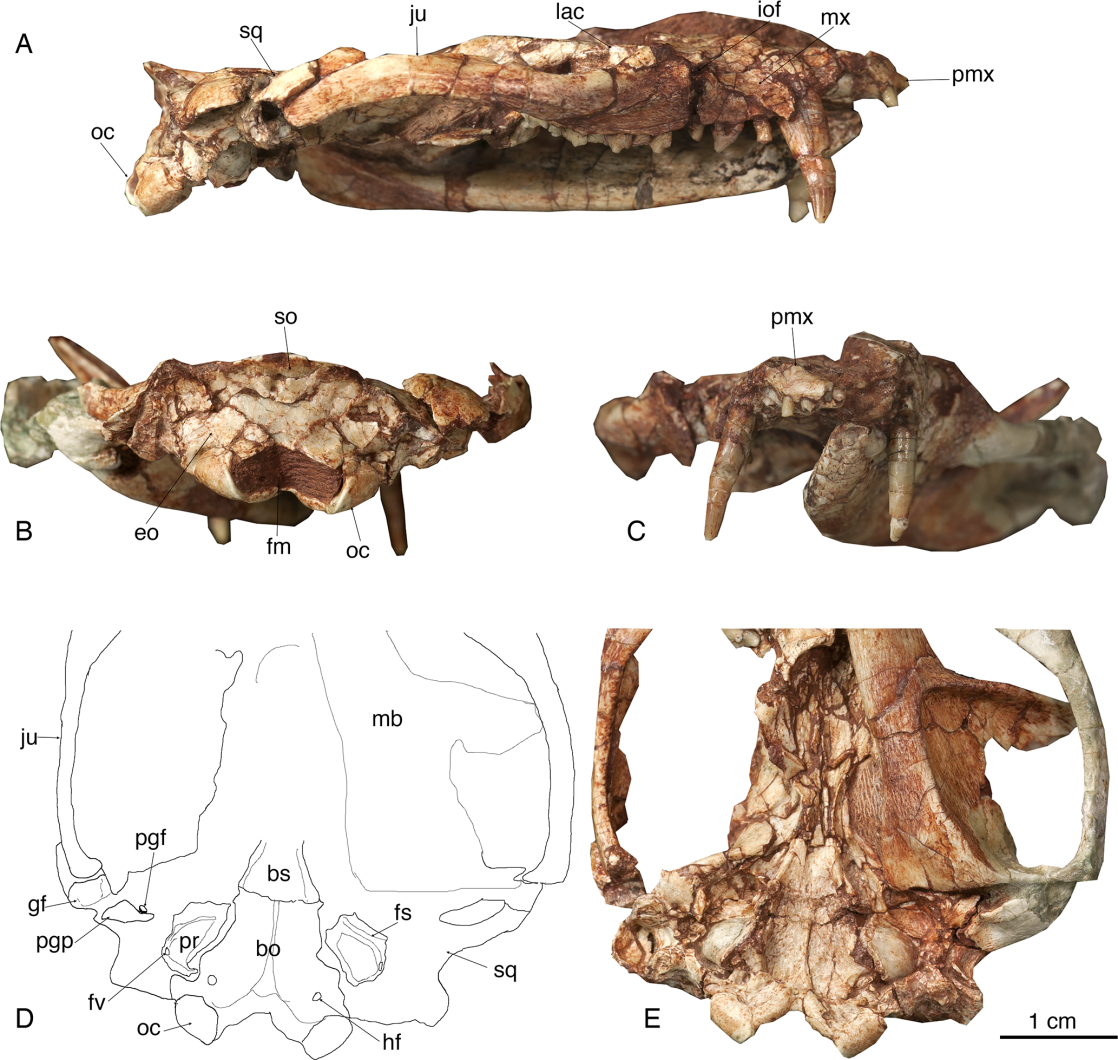
The skull of *Lotheridium mengi* (ZMNH M9032). (A) The lateral view of the right side of the skull. (B) The skull in occipital view. (C) The skull in rostral view. (D) Explanatory drawing of the basiocranium. (E) The close-up view of the basiocranium. bo, basioccipital; bs, basisphenoid; eo, exoccipital; fv, fenestra vestibuli; fm, foramen magnum; fs, facial sulcus; gf, glenoid fossa; hf, hypoglossal foramen; iof, infraorbital foramen; ju, jugal; lac, lacrimal; mb, mandible; mx, maxillae; oc, occipital condyle; pgf, postglenoid foramen; pgp, postglenoid process; pmx, premaxilla; pr, promontorium; so, supraoccipital; sq, squamosal.

**Figure 3:**
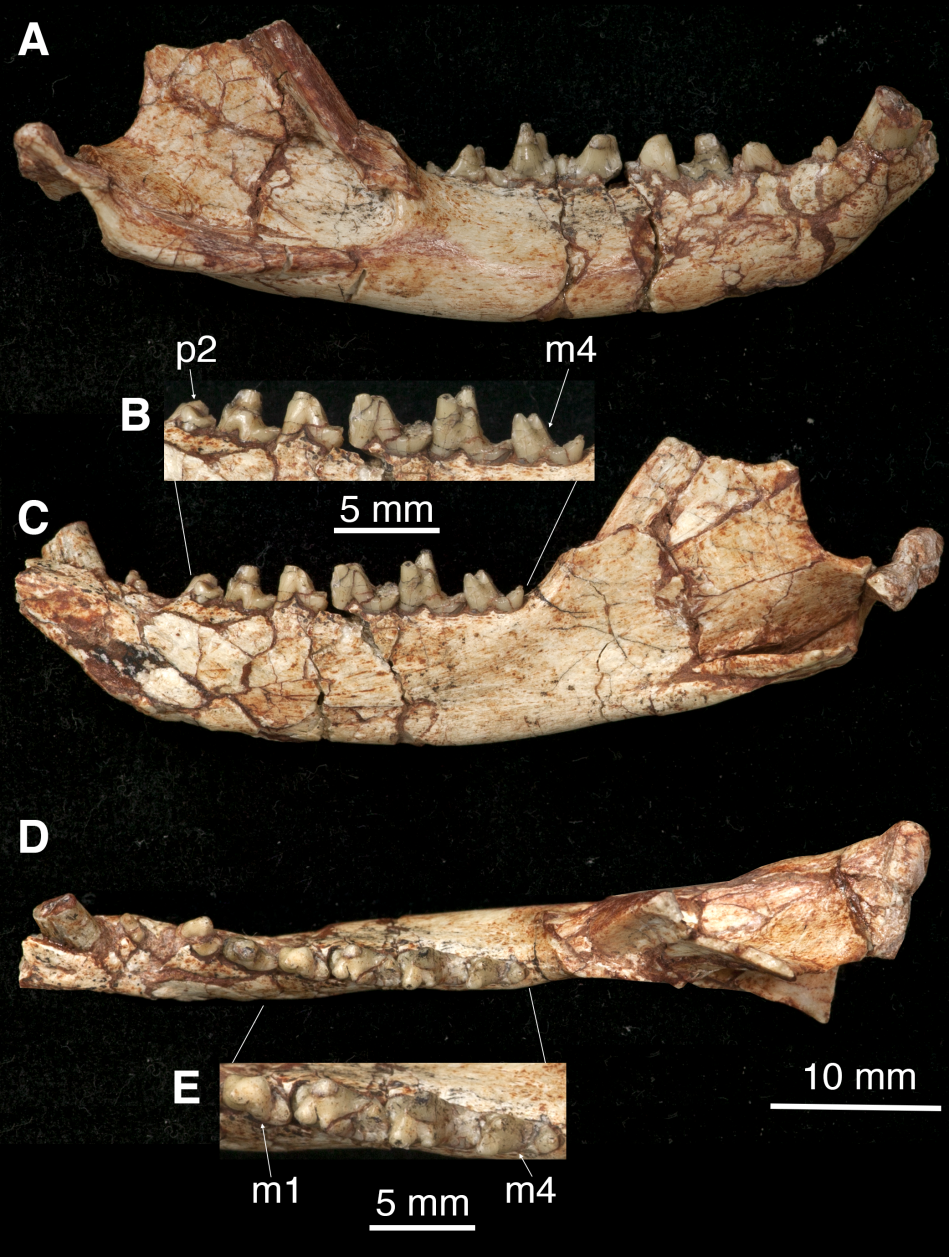
The right mandible of *Lotheridium mengi* (ZMNH M9032). (A) Lateral view. (B) Close-up view of the lingual side of p2-m4. (C) Lingual view. (D) Occlusal view. (E) Close-up view of the occlusal surface of m1-m4. Scale bar for B and E, 5 mm; Scale bar for A, C and D, 10 mm.

**Figure 4:**
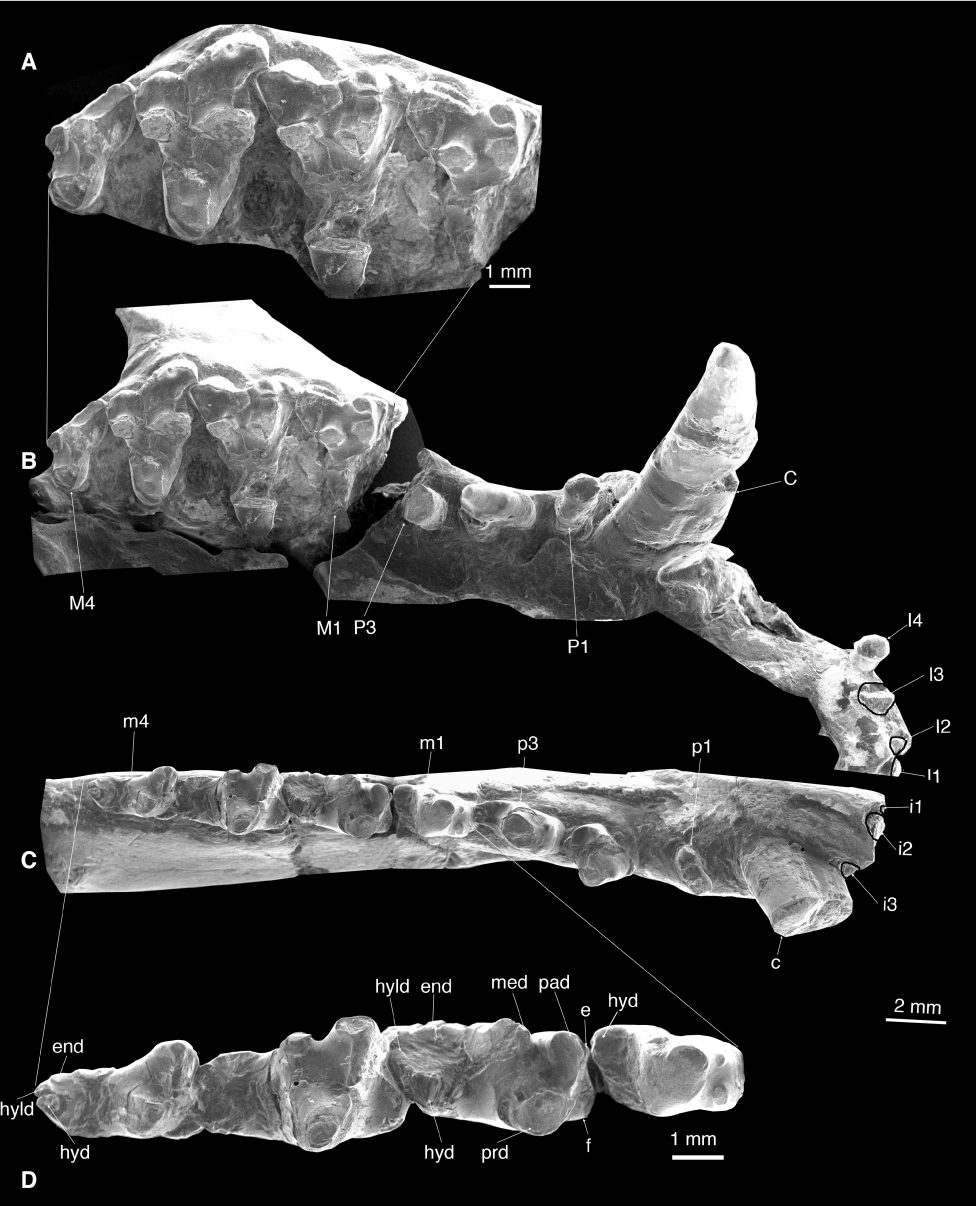
Dentition of *Lotheridium mengi* (ZMNH M9032). (A) Close-up view of M1-M4. (B) Upper dentition in occlusal view. (C) Lower dentition in occlusal view. (D) Close-up view of m1-m4. e, cuspule e; end, entoconid; f, cupsule f; hyd, hypoconid; hyld, hypoconulid; med, metaconid; pad, paraconid; prd, protoconid. Scale bar for A and D, 1 mm; Scale bar for B and C, 2 mm.

**Holotype** A nearly complete skull with associated lower jaws (ZMNH M9032, Zhejiang Museum of Natural History, Hangzhou, Zhejiang Province) (Figs. 1–4; Table 1)

**Table 1:** Measurements of teeth based on the right side (length/width in mm).

Upper teeth	Length	Width	Lower teeth	Length	Trigonid width	Talonid width
I1	0.99	0.93	i1^a^	0.53	0.60	
I2	0.85	0.64	i2^a^	1.52	1.25	
I3	1.30	1.28	i3^a^	1.20	0.90	
I4	1.27	1.00				
C	4.10	2.8	c	3.40	2.50	
P1	1.40	1.10	p1	2.00	1.03	
P2	2.82	1.40	p2	2.84	1.43	
P3^a^	3.30	2.50	p3	3.33	1.73	
M1	3.70	5.40	m1	3.34	1.80	1.09
M2	4.20	6.20	m2	4.36	2.46	1.76
M3	3.46	5.42	m3	4.40	2.58	1.73
M4	2.50	3.67	m4	3.53	1.92	1.42

**Notes.**

a P3 is broken and the measurements are estimated from the alveoli; i1-3 are based on the cross section of the tooth root because the tooth crown are all broken.

**Locality and Age** Haoping Village, Luanchuan County of Henan Province; Upper Cretaceous, Qiupa Fromation (Jiang et al., 2011).

**Etymology** The specific name is in honour of Dr. Jin Meng for his contributions to the study of Mesozoic mammals.

**Diagnosis** Dental formula: I4/3 C1/1 P3/3 M4/4. Distinctive from all other deltatheroidans in being much larger in size (except for *Delatheroides)*, in having upper molars with a more buccolingually extended protoconal region and shelf-like cuspule f on the lower molars. It differs from *Deltatheridium* and *Deltatheroides* in having upper molars with more buccally placed para-metacone and narrower stylar shelf and p1 oriented obliquely to the jaw axis. It is similar to *Deltatheridium* and differs from *Deltatheroides* in having M2 relatively larger with a deeper ectoflexus and an asymmetrical M3 with reduced metastylar lobe. It further differs from *Deltatheridium* in having the relatively unreduced last molar. It further differs from *Tsagandelta* in the absence of a sharp mesial keel below the paraconid and a mesiodistally longer and transversely wider talonid, and further differs from *Sulestes* in having upper molars with weaker ectoflexus, m4 without the metaconid, and the absence of the meckelian groove on the dentary. It further differs from *Oklatheridium* in having shallower ectoflexus, weaker postmetacrista (hypertrophied in *Oklatheridium*), weaker conular cristae and conules, lesser development of the metacone, and paraconid in a lingual position, directly mesial to the metaconid. It further differs from *Atokatheridium* in having stronger parastyle and stylocone, more developed parastylar lobe, asymmetrical M3, wider stylar shelf of upper molars, and paraconid in a lingual position, directly mesial to the metaconid. It further differs from *Nanocuris* in having lower molars with a well-developed metaconid, a proportionately large m4, and more inflated stylocone.

## Description

The skull and mandibles were originally in occlusal articulation. The right mandible was separated from the skull in preparation to expose the occlusal morphology of the dentition. The skull suffered post-mortem compression dorsoventrally so that the cranial roof is slightly crushed. The fossil was interpreted as having the derived metatherian dental formula: I4/3 C1/1 P3/3 M4/4 (More recently, the postcanine dental formula for Metatheria was interpreted as P1/p1, P2/p2, P4/p4, Dp5/dp5, M1/m1, M2/m2, and M3/m3 (O’Leary et al., 2013). However, the traditional terminology of three premolars and four molars is followed here for practical purposes). The fully erupted dentition and tooth wear indicate that the new specimen represents a fully-grown adult.

### Skull

The skull length (from the tip of the premaxilla to the posterior border of the occipital condyles) is 67.3 mm. The snout is short, less than a third of the length of the entire skull (Figs. 1A–1B and 2A). The tip of the snout has been broken and the left premaxilla is missing. The premaxilla forms the floor and lateral walls of the external nasal aperture and wedges dorsoposteriorly between the nasal and maxilla (Figs. 2A and 2C). In ventral view, the premaxilla is a short element that contains incisors I1-I4 (Figs. 1C and 4B). Its posterior border contributes to the anteromedial rim of the alveolus for the upper canine. There is a large depression for the tip of the lower canine between I4 and C. The incisive foramen is discernable medial to the alveoli of I2-4 and the depression (Fig. 1C). Most of the foramen is within the premaxilla and the maxilla forms the posterior border, as in *Monodelphis*. The paired nasal bones are parallel sided for most of their anterior length, and strongly expand posteriorly. The contact between the nasal and frontal is very broad although the nasofrontal suture cannot be ascertained due to the breakage. The maxilla contacts the nasal dorsally, the jugal and lacrimal dorsoposteriorly, but does not reach the frontal (Figs. 1A, 1B, 1D and 2A). It has a large facial process that is the major element of the lateral wall of the snout. The infraorbital foramen is large, dorsal to the posterior root of P3. The maxilla anterior to the foramen is concave, perhaps representing an attachment for the facial musculature. The maxilla contributes little to the zygomatic arch but forms the floor of the orbit. The lacrimal borders the orbit anteriorly and has a sizable expansion on the facial region (Figs. 1A–1B and 2A). It contacts the jugal posterolaterally, the maxilla anteriorly and the nasal and frontal dorsally. There are two lacrimal foramina along the anterior emargination of the orbit, separated by a distinct lacrimal tubercle. Within the orbit, the lacrimal contributes to the roof of the posterior opening of the infraorbital canal and anteromedial wall of the orbit. The anterior margin of the orbit is level with the anterior root of M1. The frontal is short and only forms the mid-section of the skull roof (Figs. 1A–1B). In dorsal view, the frontal bears a blunt postorbital process. Just posterior to the processes, the low temporal lines converge gradually posteriorly and meet at the frontal-parietal suture to form a sagittal crest. The postorbital constriction is situated at the frontal-parietal suture. In lateral view, the frontal contributes the anteromedial wall of the orbit, but its contact with other elements in the ventral region of the orbit is crushed. The parietal is large and forms the bulk of the skull roof (Figs. 1A–1B). Unlike the end-to-end interdigtated contact of the nasal and frontal, the parietal dorsally overlaps the frontal at their contact. A low sagittal crest runs from the anterior ends of the parietals to the lambdoidal crest. The parietal contacts the squamosal posteroventrally and forms the central portion of the lambdoidal crest. The jugal is robust and forms the main body of the zygoma. It extends anteriorly to contact the lacrimal and maxilla and posteriorly the jugal is dorsally overlapped by the squamosal zygoma (Figs. 1D and 2A). At the midpoint of the arched zygoma, there is a dorsal process of the jugal that marks the posteroventral border of the orbit (Fig. 1A). Its posterior end forms the anterolateral wall of the glenoid fossa (Figs. 2D–2E). The palatine is very extensive and extends far posteriorly behind the last molar and lacks palatal vacuities (Fig. 1C). The anterior extent of the palatine is unclear because of the breakage in the area. The squamosal forms the posterior sidewall of the braincase (Figs. 1A and 2A). The zygomatic process of the squamosal is short, overlying the jugal anteriorly. In ventral view, the glenoid fossa is transversely elongated, located entirely on the posterior zygomatic root (Figs. 2D–2E). Behind the fossa, there is a stout postglenoid process. The postglenoid foramen is medial to the process. The basicranium is badly fractured anteriorly; the choanal area in front of the basisphenoid can not be interpreted (Figs. 1C and 2E). The basisphenoid bone occupied the midline of the basicranium and widens posteriorly to its suture with the basiocciptal (Figs. 2D–2E). Its ventral surface is flat, flanked by rounded raised lateral ridges. The basioccipital forms the skull base and is fused with the basisphenoid anteriorly in a straight suture. Its posterior side is indented by a V-shaped intercondyloid notch. Lateral to the notch, the occipital condyles are large and protrude posteriorly. The hypoglossal foramen is located anterior to the condyle. In the middle ear region, the promontorium of the petrosal is well preserved on each side. It is bulbous-shaped and abuts to the posterolateral border of the basioccipital (Figs. 2D–2E). The anteroventral surface of the promontorium is excavated by a shallow, anteromedially directed sulcus for facial nerve. The fenestra vestibuli occupies the posterolateral corner of the promontorium and faces laterally. The surface of the promontorium is similar to that in *Deltatheridium* in lacking vascular grooves, but detailed description and comparisons await CT data. The other foramina and sulci are hard to determine because they are obliterated or masked by fractures. The supraoccipital bears a strong lambdoidal crest that projects slightly posteriorly (Fig. 2B). The exocciptials form most of the dorsolateral wall of the foramen magnum and also contribute to the dorsoposterior part of the occipital condyles.

### Mandible

The mandible is robust with a gently curved ventral outline and has its maximum depth below m3 (Figs. 3A and 3C). In the lateral view, the mandible preserves two mental foramina, one below the p2 and the other below m1 (Fig. 1D). The coronoid process is tall, its anterior edge forming a 135° angle with the tooth row. The masseteric fossa is deeply excavated with strong superior and inferior crests. It terminates anteriorly below the base of the coronoid process, well distal to the last molar (Fig. 3A). On the lingual side, the symphysis is shallow and not fused. The pterygoid fossa is shallow and extends anteriorly to the posterior root of m2. The angular process is medially inflected as seen in *Deltatheridium* and living marsupials (Fig. 3C). At the anterodorsal base of the angular process a mandibular foramen can be seen. The elongate and transversely oriented condyle is low, near the level of the tooth row (Fig. 3C).

### Upper dentition

I1-I4 are preserved on the right premaxilla (Figs. 1C and 4B; Table 1). These incisors are small and single-rooted. The crowns of I1-3 are not preserved but the roots indicate slightly procumbent and simple crowned teeth. I4 is heavily worn and pillar-like. The I1 and I2 are very closely appressed, whereas I2-I4 are separated by small gaps. Posterior to I4 is a notched diastema that accommodates the large lower canine.

The single-rooted canine is extremely long (11.9 mm) and is primarily lodged in the maxilla (Figs. 1C, 1D, 2A, 2C and 4A). It is transversely compressed and is almost perpendicular to the alveolar plane of the maxilla (Table 1).

P1 is dominated by a small conical cusp (Figs. 1C and 4B; Table 1). It is single-rooted and is directed obliquely anterolaterally. There is a diastema between P1 and P2 and between P2 and P3.

P2 has two roots and is mesiodistally long and transversely narrow (Figs. 1C and 4B; Table 1). Its crown consists of a main cusp that has a steep anterior surface and a gentle posterior surface that ends as a weak heel.

The crown of P3 is broken but the alveoli show it is double-rooted and approximately 50% larger than P2 (Figs. 1C and 4B; Table 1).

All molars are considerably worn, indicating an old individual. A sharp morphological break distinguishes the molars from the premolars. In addition, in both upper and lower dentitions, the molar wear decreases posteriorly, indicating that the molars erupted in a mesiodistal sequence. M2 is the largest upper molar (Table 1), contrasting to the condition of *Deltatheroides* in which the M1, M2, and M3 increase considerably in size distally.

The parastylar lobe of M1 is almost equally developed as the metastylar lobe, giving the crown an isosceles triangle outline (Figs. 1C, 4A and 4B; Table 1). The stylar shelf is narrow, with less lingually placed para-metacone than in *Deltatheridium*. The ectoflexus is shallow, and the ectocingulum is well developed, rimmed by crenulations. The stylocone is prominent, approximately one-half the height of the paracone and stands directly buccal to the paracone. The parastyle is small but distinct, abutting the mesial base of the stylocone. The paracone is taller and larger than the metacone, and these two cusps are joined at their base. The preparacrista is moderately developed, connected the paracone to the stylocone by a shallow notch. The postmetacrista is a strong ridge with a deep carnassial notch and directed posterobuccally. The protoconal region is missing; from what is present, it is mesiodistally short but is buccolingually extended so that the tooth is distinctively transverse and the trigon is large, approximately 60% of the crown width.

The M2 is similar to M1, but 15% larger in width (Table 1). The paracone is more prominent on the M2 than on the M1 (Figs. 4A and 4B). The protocone is moderately worn. It is tall and is more buccolingually expanded and thus M2 is transversely wider than M1 and M3. The protocristae are missing due to the breakage.

The M3 is larger than M1 but smaller than M2 (Table 1). It differs from M1-2 in having a strongly reduced metastylar but a larger parastylar lobe so that the outline of the crown in occlusal view is asymmetrical (Figs. 1C, 4A and 4B). The stylocone is large and the preparacrista runs from the paracone to the parastyle, rather than the stylocone. The ectocingulum is stronger and is rimmed by more cuspules. As with M1 and M2, the paracone is larger than the metacone, but more prominent on M3. Unlike M1 and M2, the protocone is more mesiodistally expanded but less buccolingually extended. The protocristae exhibit considerable wear, conules are lacking. The preprotocrista and postprotocrista terminate at the lingual base of the paracone and the metacone, respectively.

M4 is the smallest and most asymmetrical molar due to the lack of the metastylar lobe (Figs. 1C, 4A and 4B; Table 1). The relative development of the M4 paracone and protocone are similar to those of M3 but its metacone is very reduced. The stylocone is more mesiobuccally placed so that the preparacrista is developed as a sharp ridge, extends mesiobuccally. The parastyle is absent. The reduction of M4 is similar to that seen in *Deltatheroides*, but not significantly reduced as that in *Deltatheridium pretriberculare*.

### Lower dentition

There are three lower incisors (Fig. 4C). All lower incisors are broken; the mesial one (i1) is the smallest and the middle one (i2) is the largest (Table 1); both are closely positioned. The i3 is separated from i2 by a small gap.

The lower canine is strong and tall and is slightly more curved than its counterpart (Figs. 3A, 3C, 3D and 4C). The tip is broken. It is oval in outline (Table 1) and the tooth surfaces are gently convex mesiobuccally and flat lingually. In occlusion position, the distal portion of the lower canine rests in the notched diastema in the maxilla between the I4 and the upper canine.

All premolars are double-rooted. There is a diastema separating p1 from the preceding canine and succeeding p2, the latter one is greater. The p1 is small and positioned obliquely to the longitudinal axis of the mandible (Figs. 3A, 3C, 3D and 4C; Table 1). The crown is single-cusped and slightly procumbent with a small posterior heel.

The p2 is much larger and higher than p1 (Table 1). It is dominated by a main cusp and a transversely expanded basal heel (Figs. 3A, 3C, 3D and 4C). The main cusp has a straight mesial edge and slightly curved distal edge.

The p3 is similar to p2 but has a more developed talonid heel (Figs. 3A–3D and 4C; Table 1). There is a small but distinct cuspule at the mesiolingual base of the main cusp. The enamel of p3 has a slightly darker colour than that of the preceding two premolars, suggesting more calcification. The crown is less worn than that of anterior premolars and the succeeding m1, clearly indicating that the p3 is a successional tooth, while the p1 and p2 are members of the primary dentition (Luckett, 1993).

The m1 is considerably smaller than the succeeding m2 and m3 (Figs. 3B, 3E, 4C and 4D; Table 1). The deep wear of the tooth (especially as compared to the p3) indicates that it is not a successional tooth; the m1 in metatherians is most likely homologous with the deciduous p5 (dp5) of eutherians, as recently proposed by O’Leary et al. (2013). The trigonid is heavily worn and the cusps are not perceptible (Figs. 3E and 4D). The prominent cuspule f is shelf-like and appressed to the mesiobuccal base of the paraconid, as in *Kielantherium* (Fig. 4D; Dashzeveg & Kielan-Jaworowska, 1984). There is a small, mesiolingual projection at the base of the paraconid that we interpreted as a small cuspule e. The posterior wall of the trigonid is steeply sloped. The trigonid is considerably higher than the talonid, which is not fully developed compared to that of m2-m4 (Figs. 3A–3C). At the distal end of the talonid there is a large cusp that we interpret as the hypoconid that is displaced lingually. The hypoconulid and entoconid are not developed.

The m2-3 are similar to one another and are significantly larger than m1 (Table 1). The protoconid is the largest and positioned distobuccal to the paraconid and buccal to the metaconid (Figs. 3D, 3E, 4C and 4D). The paracristid was deeply notched and formed a carnassial shearing surface. The paraconid is slightly lower than the protoconid, but much higher than the metaconid (Figs. 3B and 4D). The metaconid is directly distal to the paraconid in the lingual position; they are not connected so the trigonid is lingually open. The cuspule f is large and shelf-like while the cuspule e is minute. The protocristid is poorly defined and has a well-developed wear facet on the posterior surface. The talonid is much lower and narrower than the trigonid. It is moderately worn but all cusps are discernable. The hypoconulid is the largest and is more on the lingual side of the talonid. The hypoconid is slightly smaller and positioned slightly mesiobuccal to the hypoconulid. The entoconid, mesiolingual to the hypoconulid, is indistinct due to the wear. On m2 there is a fracture extending from the tip of the metaconid to the hypoconid and obliterating the distal metacristid. On m3 the metaconid is strongly reduced in size, with the paraconid much higher (Figs. 3B and 4D). The distal metacristid is weakly developed.

The m4 is smaller than m2-3 (Table 1). The distinctive feature of m4 is the lack of metaconid on the trigonid (Figs. 3B, 3E, 4C and 4D). As in *Sulestes*, the talonid is much narrower and relatively longer than in m2-3. The hypoconulid is distinct and is centrally placed at the distal end of the talonid. The entoconid is minute and is separated from the hypoconulid by a shallow groove. The hypoconid is directly buccal to and is slightly larger than the hypoconulid. The distal metacristid is absent.

## Comparison

Within the Deltatheroida, *Deltatheridium* is the most completely known genus from the Upper Cretaceous Djadokhta Formation of Mongolia, represented by the skull and mandible (Gregory & Simpson, 1926; Rougier, Wible & Novacek, 1998). *Lotheridium mengi* gen. et sp. nov. closely resembles *Deltatheridium* in having a premaxilla contributing to the alveolus of the canine, a distinctive shelf-like, medially inflected angle, the absence of palatal vacuities, M2 being largest among upper molars, an asymmetrical, relatively smaller M3, a less mesiodistally expanded protocone, small p1, and m4 with double cusped trigonid and vestigial talonid. However, it differs from *Deltatheridium* in being much larger, upper molars with a more buccolingually expanded protoconal region and narrower stylar shelf, the absence of conules, M4 not significantly reduced, obliquely oriented p1, and the larger size of the cuspule f on lower molars.

*Deltatheroides* was also recovered from the Upper Cretaceous Djadokhta Formation of Mongolia (Gregory & Simpson, 1926; Kielan-Jaworowska, 1975). *Lotheridium mengi* differs from *Deltatheroides* in having M1-M3 not increasing in size, an asymmetrical M3, obliquely oriented p1, m4 having an incompletely developed trigonid with the absence of metaconid and a reduced talonid, and the absence of palatal vacuities.

*Tsagandelta dashzevegi* has also been described from the Upper Cretaceous Baynshiree Formation of eastern Mongolia, known from a partial dentary with m2-m3 (Rougier, Davis & Novacek, 2015). It is similar to *Lotheridium mengi* in having a robust dentary and the presence of cuspule f, but differs in being smaller in size and having a mesiodistally shorter and transversely narrower talonid, the development of a sharp mesial keel below the paraconid, and the lack of cuspule e.

*Sulestes karakshi* was recovered form the Upper Cretaceous Bissekty fauna of Uzbekistan (Averianov, Archibald & Ekdale, 2010; Nessov, 1985). Two lower molars and an edentulous fragment of a maxilla from the Bissekty Formation of Uzbekistan were initially described by Nessov (1997) as *Deltatheroides kizylkumensis* and later transferred to its own genus, *Deltatherus*(Nessov, 1997). Subsequently, these specimens were referred to *Sulestes karakshi* by Averianov, Archibald & Ekdale (2010). *Sulestes* is similar to *Lotheridium mengi* by an absence of palatal vacuities, anterior wall of the upper canine alveolus formed by premaxilla, an asymmetrical M3 with reduced metastylar lobe, an unreduced M4, and obliquely oriented p1. However, *Sulestes* differs from *Lotheridium mengi* in being much smaller in size, having better developed conules and internal conular cristae on upper molars, double-rooted P1, m4 with metaconid, and the presence of Meckelian groove on the dentary.

The North American Early Cretaceous *Atokatheridium* and *Oklatheridium* have been confidently referred to the Deltatherididae (Davis, Cifelli & Kielan-Jaworowska, 2008; Davis & Cifelli, 2011). *Lotheridium mengi* resembles *Atokatheridium* and *Oklatheridium* in the plesiomorphic presence of cuspule e and f on the trigonid, a condition also present on early boreosphenidans. However, *Oklatheridium* differs from *Lotheridium mengi* in having a deep ectoflexus, lesser width of the protoconal region, well-developed conules and conular cristae, and the metacone broader than the protocone. *Lotheridium mengi* differs from *Atokatheridium* in being larger in size, in having larger stylocone, more developed metastylar lobe and parastylar lobe on M1 and M2, and a less development metastylar lobe on M3.

*Nanocuris improvida* from the Late Cretaceous of Canada was originally placed in its own family, Nanocuridae, within Eutheria (Fox, Scott & Bryant, 2007). Based on the Lancian specimens of Wyoming, Wilson & Riedel (2010) subsumed it within the family Deltatheridiidae. It differs from *Lotheridium mengi* in having upper molars with a mesiodistally longer and transversely narrower protoconal region, upper molar with a weaker stylocone, lower molars with a highly reduced metaconid, and the more developed distal metacristid on lower molars.

### Phylogenetic analysis

To assess the phylogenetic position of *Lotheridium*, we performed a phylogenetic analysis using the data matrix of Rougier, Davis & Novacek (2015) with addition of new taxon. This matrix was modified and expanded from three recent studies (Averianov, Archibald & Ekdale, 2010; Rougier, Wible & Novacek, 2004; Wilson & Riedel, 2010), primarily from Rougier, Wible & Novacek (2004). Taxa (*Aegialodon*, *Comanchea*, *Trinititherium, Falepetrus*, *Zygiocuspis*, and *Slaughteria*) have been excluded from the analysis either because the fossils are known by a single tooth or because there is alternative interpretation of the tooth loci (Rougier, Davis & Novacek, 2015). Appendix 1 provides scores for *Lotheridium*.

**Figure 5:**
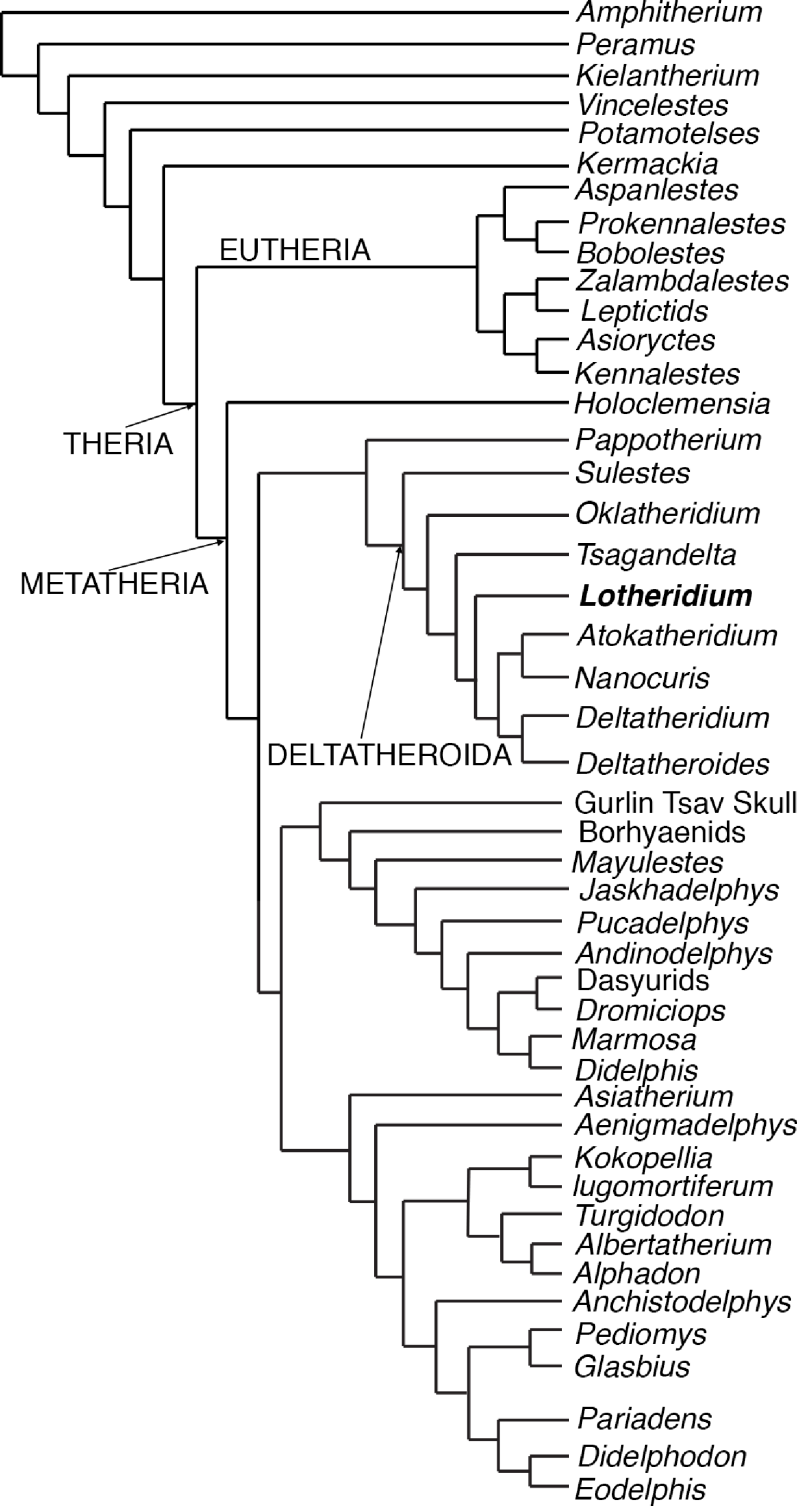
The most parsimonious tree generated from the phylogenetic analyses from this paper. The cladistics analysis is based on 46 taxa and 156 craniodental characters.

We performed a ratchet search under New Technology menu of TNT (version 1. 1 by Pablo Goloboff, Steve Farris, and Kevin Nixon). We followed Rougier, Davis & Novacek (2015) in designating 11 multiple state characters as ordered (1, 4, 7, 12, 14, 35, 36, 50, 51, 52, 116); the remaining characters were unordered. The analysis generated only one most parsimonious tree (Fig. 5) with a tree length of 559 steps, a consistency index of 0.37, and a retention index of 0.67.

## Results and Discussion

Our most parsimonious tree (Fig. 5) is nearly identical to that of Rougier, Davis & Novacek (2015) except for the position of *Oklatheridium*. In our tree topology, *Oklatheridium* is intermediate between *Sulestes* and other deltatheroidans while *Oklatheridium* forms a monophyletic clade with *Sulestes* in Rougier, Davis & Novacek (2015)’s tree. *Lotheridium mengi* is deeply nested within deltatheroidans and shares with other deltatheroidans the following derived features: the metastylar area on penultimate upper molar reduced, the parastyle small or indistinct, the absence of the metasyle, the metacone and paracone bases confluent, salient postmetacrista strongly developed, last upper molar reduced. Specifically, it is more derived than *Sulestes*, *Oklatheridium*, and *Tsagandelta*, but more plesiomorphic than the clade (*Deltatheridium* + *Deltatheroides*) and (*Atokatheridium* + *Nanocuris*).

It has been proposed that the distinctive marsupial tooth-replacement pattern, in which only the deciduous P3/p3 are replaced and the deciduous P1-2/p1-2 are retained throughout life, was present in *Deltatheridium* (Rougier, Wible & Novacek, 1998). The pattern, also present in living marsupials and several Cretaceous-Paleocene metatherian taxa, has been considered highly diagnostic for metatherians (Cifelli & Muizon, 1998; Luckett, 1993). This determination, based on a juvenile specimen of *Deltatheridium pretrituberculare* (PSS–MAE 132) (Rougier, Wible & Novacek, 1998), has been challenged by Kielan-Jaworowska, Cifelli & Luo (2004: 438), who stated: “Given the relatively advanced ontogenetic age of the specimen, homologies of the teeth (deciduous or replaced) at the p1-2 loci are uncertain….” Fox & Naylor (2006: 27) further argued: “From the actual evidence that PSS-MAE 132 presents, however, the only valid conclusion that can be drawn is that the teeth in place at the p1–2 loci had erupted earlier than that at the p3 position…” In contrast, *Lotheridium mengi* is from an adult individual. The crowns of p1 and p2 exhibit substantial wear, whereas that of p3 only shows slight wear (Figs. 3D, 4C and 4D). The base of the anterior two premolars from the alveolar border of the dentary erupted much higher than that of the ultimate premolar and the molars, implying that the anterior two premolars erupted considerably earlier than p3 and the molars (Luckett, 1993). It is unlikely that the anterior two premolars had predecessors; they represent teeth from the primary dentition. Moreover, wear patterns suggest the p3 erupted about the same time as the m4 as in some living marsupials and *Alphadon* (Cifelli et al., 1996). This strongly corroborates Rougier, Wible & Novacek (1998)’s hypothesis that the distinctive marsupial tooth-replacement pattern, in which only the deciduous P3/p3 had two generations throughout life, was already present in deltatheroidans. This tooth replacement pattern again supports a marsupial affinity for deltatheroidans.

Moreover, *Lotheridium mengi* resembles *Deltatheridium*, but differs from Mesozoic outgroups used in this study, monotremes and eutherians, in having many metatherian features: notably, a distinctive shelf-like, medially inflected angle, and marsupial dental formula of three premolars and four molars. These features, coupled with the tooth replacement pattern, indicate that deltatheroidans may have developed a feeding system similar to that of marsupials, which further implies the possibility that they already possessed the basic marsupial reproductive pattern.

Deltatheroida have traditionally been thought to originate in Asia (Cifelli, 2000; Kielan-Jaworowska & Nessov, 1990) because species of the group were predominantly recovered from Asia. In addition, *Sulestes* from the Coniacian of Uzbekistan has been regarded as the oldest uncontested taxon among deltatheroidans. Yet, fossils from North America have sparked a paleobiogeographic hypothesis that favors a North American origin of the clade. *Atokatheridium* and *Oklatheridium* from the Upper Cretaceous of southern Oklahoma were referred to Deltatheroida (Davis, Cifelli & Kielan-Jaworowska, 2008; Davis & Cifelli, 2011; Kielan-Jaworowska & Cifelli, 2001). Because these North American species are significantly older than *Sulestes*, some researchers proposed a North American origin for deltatheroidans (Davis, Cifelli & Kielan-Jaworowska, 2008; Rougier, Wible & Novacek, 2004). However, Wilson & Riedel (2010) argued that Asian species, although younger, are relatively primitive and were commonly placed as basal members of deltatheroidans in phylogenetic analyses. Thus, these authors suggested that Deltatheroida originated in Asia and then followed by a single dispersal event to North America by the Aptian-Albian. More recently, Rougier, Davis & Novacek (2015) proposed two alternate models for the origin of the deltatheroidans: one assumes that parallel adaptive radiation have occurred within the deltatheroidans on each continent, with the most derived taxa clustering geographically (a North American clade and an Asian clade), while the other suggests the possibility of multiple dispersal events between Asia and North America. Our phylogenetic analysis suggests that *Sulestes* was  more plesiomorphic than other deltatheroidans, corroborating previous suggestions of an Asiatic origin for the clade (Wilson & Riedel, 2010). *Lotheridium mengi* was recovered from Upper Cretaceous rocks of Henan Province, China, which does not provide information on where or when deltatheroidans originated. However, along with the fossil records in Uzbekistan and Mongolia of central Asia, the new species does suggest that deltatheroidans were more diverse and widely distributed in Asia than previously thought (Meng, 2014).

## APPENDIX 1

The scoring for *Lotheridium mengi*:

**Table:**

2001021111	2000111112	0000221100	0100020001	1111100010	0101010000	110?111001
1011111?11	0100100001	00000000??	??????????	??1110?1?1	??22??????	???000????
??????????	???001.

## Additional Information and Declarations

### New Species Registration

The following information was supplied regarding the registration of a newly described species: The Life Science Identifiers (LSIDs) for this publication include: urn:lsid:zoobank.org:pub:5FE0ADE4-3EFD-488B-AC21-D7278AF7E514 urn:lsid:zoobank.org:act:0691BE9D-0A73-42CA-896B-DF77290838C1 (genus) urn:lsid:zoobank.org:act:34627187-29BC-435D-B2BE-EB5C439B5020 (species).

## References

[ref-1] Averianov AO, Archibald JD, Ekdale EG (2010). New material of the Late Cretaceous deltatheroidan mammal *Sulestes* from Uzbekistan and phylogenetic reassessment of the metatherian-eutherian dichotomy. Journal of Systematic Palaeontology.

[ref-2] Butler PM, Kielan-Jaworowska Z (1973). Is *Deltatheridium* a marsupial?. Nature.

[ref-3] Cifelli RL (1993a). Early Cretaceous mammal from North America and the evolution of marsupial dental characters. Proceedings of the National Academy of Sciences of the United States of America.

[ref-4] Cifelli RL, Szalay FS, Novacek MJ, McKenna MC (1993b). Theria of metatherian-eutherian grade and the origin of marsupials. Mammal Phylogeny, Volume 2—Mesozoic differentiation, multituberculates, monotremes, early therians, and marsupials.

[ref-5] Cifelli RL (2000). Cretaceous mammals of Asia and North America. Paleontological Society of Korea Special Publication.

[ref-6] Cifelli RL, Muizon CD (1998). Tooth eruption and replacement pattern in early marsupials. Comptes Rendus de l’Académie des Sciences, Sciences de la terre et des planètes, Paris.

[ref-7] Cifelli RL, Rowe TB, Luckett WP, Banta J, Reyes R, Howes RI (1996). Fossil evidence for the origin of the marsupial pattern of tooth replacement. Nature.

[ref-8] Dashzeveg D, Kielan-Jaworowska Z (1984). The lower jaw of an aegialodontid mammal from the Early Cretaceous of Mongolia. Zoological Journal of the Linnean Society.

[ref-9] Davis BM, Cifelli RL (2011). Reappraisal of the tribosphenidan mammals from the Trinity Group (Aptian-Albian) of Texas and Oklahoma. Acta Palaeontologica Polonica.

[ref-10] Davis BM, Cifelli RL, Kielan-Jaworowska Z, Sargis EJ, Dagosto M (2008). Earliest evidence of Deltatheroida (Mammalia: Metatheria) from the Early Cretaceous of North America. Mammalian evolutionary morphology: a tribute to Frederick S Szalay.

[ref-11] Fox RC (1974). *Deltatheroides*-like mammals from the Upper Cretaceous of North America. Nature.

[ref-12] Fox RC (1975). Molar structure and function in the Early Cretaceous mammal *Pappotherium*: evolutionary implications for Mesozoic Theria. Canadian Journal of Earth Sciences.

[ref-13] Fox RC, Naylor BG (2006). Stagodontid marsupials from the Late Cretaceous of Canada and their systematic and functional implications. Acta Palaeontologica Polonica.

[ref-14] Fox RC, Scott CS, Bryant HN (2007). A new, unusual therian mammal from the Upper Cretaceous of Saskatchewan, Canada. Cretaceous Research.

[ref-15] Gregory WK, Simpson GG (1926). Cretaceous mammal skulls from Mongolia. American Museum Novitates.

[ref-16] Jiang X, Liu Y, Ji S, Zhang X, Xu L, Jia S, Lü J, Yuan C, Li M (2011). Dinosaur-bearing strata and K/T boundary in the Luanchuan-Tantou Basin of western Henan Province, China. Science China Earth Sciences.

[ref-17] Kielan-Jaworowska Z (1975). Evolution of the therian mammals in the Late Cretaceous of Asia. Part I. Deltatheridiidae. Palaeontologia Polonica.

[ref-18] Kielan-Jaworowska Z, Cifelli RL (2001). Primitive boreosphenidan mammal (?Deltatheroida) from the Early Cretaceous of Oklahoma. Acta Palaeontologica Polonica.

[ref-19] Kielan-Jaworowska Z, Cifelli RL, Luo Z-X (2004). Mammals from the age of dinosaurs: origins, evolution, and structure.

[ref-20] Kielan-Jaworowska Z, Eaton JG, Bown TM, Lillegraven JA, Kielan-Jaworowska Z, Clemens WA (1979). Theria of metatherian-eutherian grade. Mesozoic mammals: the first two-thirds of mammalian history.

[ref-21] Kielan-Jaworowska Z, Nessov LA (1990). On the metatherian nature of the Deltatheroida, a sister group of the Marsupialia. Lethaia.

[ref-22] Luckett WP, Szalay FS, Novacek MJ, McKenna MC (1993). An ontogenetic assessment of dental homologies in therian mammals. Mammal phylogeny, volume 1 mesozoic differentiation, multituberculates, monotremes, early therians, and marsupials.

[ref-23] Luo Z-X, Ji Q, Wible JR, Yuan C-X (2003). An Early Cretaceous tribosphenic mammal and metatherian evolution. Science.

[ref-24] Marshall LG, Kielan-Jaworowska Z (1992). Relationships of the dog-like marsupials, deltatheroidans and early tribosphenic mammals. Lethaia.

[ref-25] McKenna MC, Mellett JS, Szalay FS (1971). Relationships of the Cretaceous mammal *Deltatheridium*. Journal of Paleontology.

[ref-26] Meng J (2014). Mesozoic mammals of China: implications for phylogeny and early evolution of mammals. National Science Review.

[ref-27] Muizon C, Lange-Badré B (1997). Carnivorous dental adaptations in tribosphenic mammals and phylogenetic reconstruction. Lethaia.

[ref-28] Nessov LA (1985). Rare bony fishes, terrestrial lizards and mammals from the zone of estuaries and coastal plains of the Cretaceous of Kyzylkum. Ezhegodnik Vsesoyuznogo Paleontologicheskogo Obshchestva.

[ref-29] Nessov LA (1997). Cretaceous non-marine vertebrates of northern Eurasia (in Russian).

[ref-30] O’Leary MA, Bloch JI, Flynn JJ, Gaudin TJ, Giallombardo A, Giannini NP, Goldberg SL, Kraatz BP, Luo Z-X, Meng J, Ni X, Novacek MJ, Perini FA, Randall ZS, Rougier GW, Sargis EJ, Silcox MT, Simmons NB, Spaulding M, Velazco PM, Weksler M, Wible JR, Cirranello AL (2013). The placental mammal ancestor and the post–K-Pg radiation of placentals. Science.

[ref-31] Rougier GW, Davis BM, Novacek MJ (2015). A deltatheroidan mammal from the Upper Cretaceous Baynshiree Formation, eastern Mongolia. Cretaceous Research.

[ref-32] Rougier GW, Wible JR, Novacek MJ (1998). Implications of *Deltatheridium* specimens for early marsupial history. Nature.

[ref-33] Rougier GW, Wible JR, Novacek MJ (2004). New specimen of *Deltatheroides cretacicus* (Metatheria, Deltatheroida) from the Late Cretaceous of Mongolia. Bulletin of the Carnegie Museum of Natural History.

[ref-34] Sánchez-Villagra M, Ladevèze S, Horovitz I, Argot C, Hooker JJ, Macrini TE, Martin T, Moore-Fay S, Muizon C, Schmelzle T, Asher RJ (2007). Exceptionally preserved North American Paleogene metatherians: adaptations and discovery of a major gap in the opossum fossil record. Biology Letters.

[ref-35] Simpson GG (1928). Affinities of the Mongolian Cretaceous insectivores. American Museum Novitates.

[ref-36] Szalay FS (1994). Evolutionary history of the marsupials and an analysis of osteological characters.

[ref-37] Van Valen L (1966). Deltatheridia, a new order of mammals. Bulletin of the American Museum of Natural History.

[ref-38] Williamson TE, Brusatte SL, Wilson GP (2014). The origin and early evolution of metatherian mammals: the Cretaceous record. ZooKeys.

[ref-39] Wilson GP, Riedel JA (2010). New specimen reveals deltatheroidan affinities of the North American Late Cretaceous mammal Nanocuris. Journal of Vertebrate Paleontology.

